# Reprogramming energy metabolism and inducing angiogenesis: co-expression of monocarboxylate transporters with VEGF family members in cervical adenocarcinomas

**DOI:** 10.1186/s12885-015-1842-4

**Published:** 2015-11-02

**Authors:** Céline Pinheiro, Eduardo A. Garcia, Filipa Morais-Santos, Marise A. R. Moreira, Fábio M. Almeida, Luiz F. Jubé, Geraldo S. Queiroz, Élbio C. Paula, Maria A. Andreoli, Luisa L. Villa, Adhemar Longatto-Filho, Fátima Baltazar

**Affiliations:** 1 Life and Health Sciences Research Institute (ICVS), School of Health Sciences, University of Minho, Braga, 4710-057 Portugal; 2 ICVS/3B’s - PT Government Associate Laboratory, Braga/Guimarães, Portugal; 3 Barretos School of Health Sciences, Dr. Paulo Prata – FACISB, Barretos, São Paulo Brazil; 4 Molecular Oncology Research Center, Barretos Cancer Hospital, Barretos, Sao Paulo Brazil; 5 Department of Pathology of the School of Medicine of the Federal University of Goiás, Goiânia, Go Brazil; 6 Hospital Araújo Jorge, Goiânia, Go Brazil; 7 Instituto Nacional de Ciência e Tecnologia do HPV (INCT-HPV), Sao Paulo, Brazil; 8 Department of Radiology, Center on Translational Oncology Investigation, São Paulo State Cancer Institute, Faculdade de Medicina, Universidade de São Paulo, São Paulo, Brazil; 9 Santa Casa de São Paulo Medical School, São Paulo, Brazil; 10 Laboratory of Medical Investigation (LIM-14), School of Medicine, University of Sao Paulo, Sao Paulo, Brazil

**Keywords:** Angiogenesis, Cervical adenocarcinoma, HPV, Hypoxia, Lymphangiogenesis, Metabolic reprogramming, Monocarboxylate transporter, VEGF

## Abstract

**Background:**

Deregulation of cellular energetic metabolism was recently pointed out as a hallmark of cancer cells. This deregulation involves a metabolic reprogramming that leads to a high production of lactate. Lactate efflux, besides contributing for the glycolytic flux, also acts in the extracellular matrix, contributing for cancer malignancy, by, among other effects, induction of angiogenesis. However, studies on the interplay between cancer metabolism and angiogenesis are scarce. Therefore, the aim of the present study was to evaluate the metabolic and vascular molecular profiles of cervical adenocarcinomas, their co-expression, and their relation to the clinical and pathological behavior.

**Methods:**

The immunohistochemical expression of metabolism-related proteins (MCT1, MCT4, CD147, GLUT1 and CAIX) as well as VEGF family members (VEGF-A, VEGF-C, VEGF-D, VEGFR-1, VEGFR-2 and VEGFR-3) was assessed in a series of 232 cervical adenocarcinomas. The co-expression among proteins was assessed and the expression profiles were associated with patients’ clinicopathological parameters.

**Results:**

Among the metabolism-related proteins**,** MCT4 and CAIX were the most frequently expressed in cervical adenocarcinomas while CD147 was the less frequently expressed protein. Overall, VEGF family members showed a strong and extended expression with VEGF-C and VEGFR-2 as the most frequently expressed and VEGFR-1 as the less expressed member. Co-expression of MCT isoforms with VEGF family members was demonstrated. Finally, MCT4 was associated with parametrial invasion and HPV18 infection, CD147 and GLUT1 with distant metastasis, CAIX with tumor size and HPV18 infection, and VEGFR-1 with local and lymphnode metastasis.

**Conclusions:**

The results herein presented provide additional evidence for a crosstalk between deregulating cellular energetics and inducing angiogenesis. Also, the metabolic remodeling and angiogenic switch are relevant to cancer progression and aggressiveness in adenocarcinomas.

## Background

Human papillomavirus (HPV) is a well-recognized causal agent for cervical cancer development as well as for anal, head and neck, penile cancers, among other malignancies [1]. Cervical cancer is a serious problem for developing countries’ public health authorities worldwide, due to the high annual rates of incidence and mortality. Despite several efforts in introducing screening programs, poor countries fail to reduce the effects of cervical cancer due to innumerous problems related to cultural aspects, lack of human resources and infrastructure, lack of political commitment and limited economic investments [2]. HPV vaccination is a promising tool to reduce the mortality and iniquity that is related to HPV-induced cancer, but the impact of primary prevention is a long-term investment and the costs are still over the possibility to be effectively implemented in underserved regions [3]. The last Globocan publication from the International Agency for Research on Cancer still pointed out cervical cancer as the 3^rd^ more frequent malignancy in developing countries. There are almost 500,000 new cases/year and 250,000 deaths in the World; circa 80 % of all cases occurs in developing/poor countries [4].

The development of cervical cancer from HPV infection is frequently an enduring process that takes more than ten years and involves different molecular pathways to effectively occur [5]. Successful screening programs reduced the incidence of squamous cell carcinoma, the most frequent cervical cancer histological subtype, but not of cervical adenocarcinoma, less frequent, which incidence is constantly rising. This is still a worrying question to be solved because cervical adenocarcinoma have a more aggressive biological behavior as compared with squamous cell carcinoma, with poorer prognosis and decreased survival rates. Part of these differences is attributed to distinctive molecular patterns but this topic is not fully understood [6].

The deregulation of cellular energetic metabolism was recently pointed out as a hallmark of cancer cells. This deregulation involves metabolic reprogramming where cancer cells, even in the presence of oxygen, largely limit their energetic metabolism to glycolysis, followed by pyruvate conversion into lactate (known as the Warburg effect), instead of pyruvate utilization in the mitochondria for Krebs cycle followed by oxidative phosphorylation, a much more energetically favorable metabolic pathway [7]. To allow continuous glycolytic flux, cancer cells must promote the efflux of the accumulating lactate. As a result, cancer cells upregulate the plasma membrane transporters responsible for lactate efflux—monocarboxylate transporters (MCTs) [8]. MCTs are a family of 14 members but only isoforms 1–4 transport monocarboxylates, including lactate, coupled with a proton [9]. In this context, MCT1 and MCT4, along with their chaperone CD147 [10], emerge as key proteins in the metabolic reprogramming of cancer cells, being pointed out as potential therapeutic targets for cancer therapy [8, 11, 12]. Other important proteins play key roles in the metabolic reprogramming of cancer cells, including the glucose transporter 1 (GLUT1) and the pH regulator carbonic anhydrase IX (CAIX) [12].

Lactate efflux, besides contributing to the glycolytic flux for energy and anabolic intermediate production, also detains an important role in the extracellular matrix, contributing to cancer malignancy [13]. Actually, another hallmark of cancer, induction of angiogenesis, is included in the effects of lactate in the extracellular matrix [7]. The basis for this induction is a lactate-induced secretion of vascular endothelial growth factor (VEGF), which induces angiogenesis [13]. In angiogenesis, new blood vessels grow from pre-existing endothelial cells providing the substrate necessary for cancer cells’ growth and spread [14]. VEGF, also know as VEGF-A, can induce angiogenesis in physiological and pathological conditions [15], and is the most well know member of a family composed also of VEGF-B, VEGF-C, VEGF-D and placental growth factor (PlGF) [16]. This group of highly conserved factors regulates vasculogenesis, hematopoiesis, angiogenesis, lymphangiogenesis and vascular permeability [17, 18]. Different members of this growth factor family have distinct biological function, different receptors and diverse expression patterns [19]. The family also includes three tyrosine kinase receptors: VEGFR-1 (Flt-1), VEGFR-2 (KDR) and VEGFR-3 (Flt-4). VEGF-A binds to VEGFR-1 (vascular endothelial growth factor receptor 1) and VEGFR-2, VEGF-B binds to VEGFR-1, and both VEGF-C and VEGF-D bind to VEGFR-2 and VEGFR-3 [18, 20]. Nowadays, the VEGF family members and their receptors are considered important therapeutic targets [21, 22] and increased expression of these proteins in tumors has been associated with poor prognosis and increased risk of recurrence or metastasis in several types of cancers [23, 24]. Currently, several pro- or anti-angiogenic drugs have been approved by the FDA or are in clinical trials [25].

Since studies on the interplay between cancer metabolism and angiogenesis are scarce, the aim of the present study was to evaluate the metabolic and vascular molecular profiles of cervical adenocarcinomas and the possible crosstalk between these two hallmarks of cancer. For that, the immunohistochemical expression of a variety of metabolism-related proteins and VEGF family members was evaluated in a series of cervical adenocarcinomas, the possible co-expression between metabolism-related proteins and VEGF family members was tested and the expression profiles was associated with the clinicopathological tumor behavior.

## Methods

### Human cervical adenocarcinoma samples

The series analyzed included 232 formalin-fixed paraffin-embedded cervical adenocarcinomas, retrieved from the files of Araújo Jorge Hospital and the Pathology Department of the School of Medicine of the Federal University of Goiás, Goiania, in Goiás State, Brazil. Samples were organized into tissue microarrays (TMA), ranging from 262 to 320 tumor cores (0.6 mm diameter each), also including several control samples (normal kidney and placenta). Each case was represented in the TMA by at least two cores. Clinicopathological data included age at diagnosis (mean 48 years), tobacco use, FIGO classification, tumor grade, tumor size, parametrial invasion, local, lymphnode and distant metastasis, and survival. Detailed information about the clinicopathological data is presented in Table 1. The present study was approved by the hospital ethics committee “Comitê de Ética em Pesquisa do Hospital das Clínicas” of the Federal University of Goiás”, ref. 050/2011. Since this was a retrospective study with minimal risk to the participants (characterized by the breach of confidentiality), no patient written consent was obtained but patient's identity was protected.

**Table 1:** Clinicopathological data of the cervical adenocarcinoma patients

Variable	n	%
*Tobacco use* (*n =* 117)		
No	83	70.9
Yes	34	29.1
*FIGO* (*n =* 93)		
I	49	52.7
II + III	44	47.3
*Grade* (*n =* 201)		
Low grade (I)	118	58.7
High grade (II e III)	83	41.3
*Tumor size* (*n =* 36)		
<4 cm	27	75.0
>4 cm	9	25.0
*Parametrial invasion* (*n =* 51)		
Absent	21	41.2
Present	30	58.8
*Local metastasis* (*n =* 98)		
Absent	73	74.5
Present	25	25.5
*Lymphnode metastasis* (*n =* 81)		
Absent	68	84.0
Present	13	16.0
*Distant metastasis* (*n =* 126)		
Absent	107	84.9
Present	19	15.1
*HPV type* (*n =* 208)		
Negative	45	21.6
16	98	47.1
18	34	16.3
16, 18	7	3.4
45	6	2.9
31	3	1.4
39	3	1.4
58	2	1.0
18, 45	2	1.0
11	1	0.5
16, 45, 52	1	0.5
16, 18, 11	1	0.5
16, 74	1	0.5
16, 52	1	0.5
16, 11, 52	1	0.5
16, 45	1	0.5
16, 39	1	0.5

### HPV genotyping

DNA from archived formalin-fixed paraffin-embedded samples was extracted with xylene, followed by two washes with ethanol 100 % and 50 %, respectively, and the dried pellet was incubated with 300 μl proteinase-K, overnight, at 56 °C. After purification with fenol:chloroform:isoamylic alcohol (25:24:1), the DNA was recovered and diluted in water, and concentration determined in Nanodrop 2000 (Thermo Scientific, Waltham, MA, USA). DNA quality was accessed by globin PCR using PCO3/PCO4 primers, according to Saiki et al. [26]. HPV genotyping was based on the L1 region of HPV genome, with SPF10 primers from INNO-LiPA-genotyping assay (Innogenetics, Ghent, Belgium) that identify 28 different genotypes. Results are presented in Table 1.

### Immunohistochemistry

MCT1 and CD147 immunohistochemistry was performed according to the avidin-biotin-peroxidase complex method (R.T.U. VECTASTAIN Elite ABC Kit (Universal), Vector Laboratories, Burlingame, CA, USA), as previously described [27]. Immunohistochemistry for MCT4, GLUT1 and CAIX was performed according to the streptavidin-biotin-peroxidase complex principle (Ultravision Detection System Anti-polyvalent, HRP, Lab Vision Corporation, Fremont, CA, USA), as previously described [28, 29]. Appropriate serum controls were used as negative controls (N1698 and N1699, Dako, Carpinteria, CA, USA). Colon carcinoma tissue was used as positive control for MCT1, MCT4 and CD147, head and neck cancer was used for GLUT1 and normal stomach was used for CAIX. Tissue sections were counterstained with hematoxylin and permanently mounted. Please refer to Table 2 for detailed aspects about each antibody used.

**Table 2:** Detailed aspects for each antibody used in immunohistochemistry

Protein	Antigen retrieval	Antibody	Antibody dilution and incubation time
MCT1	Citrate buffer (0.01 M, pH = 6), 98 °C, 20′	AB3538P	1:200, overnight
		Chemicon International	
MCT4	Citrate buffer (0.01 M, pH = 6), 98 °C, 20′	sc-50329	1:500, 2 h
		Santa Cruz Biotechnology	
CD147	EDTA (1 mM, pH = 8), 98 °C, 20′	sc-71038	1:400, overnight
		Santa Cruz Biotechnology	
GLUT1	Citrate buffer (0.01 M, pH = 6), 98 °C, 20′	ab15309-500	1:500, 2 h
		AbCam	
CAIX	Citrate buffer (0.01 M, pH = 6), 98 °C, 20′	ab15086	1:2000, 2 h
		AbCam	
VEGF-A	CC1 (pH = 8,2) Ventana	VG-1	1:200, 60 min
		AbCam	
VEGF-C	ptLink (pH = 9) Dako	18-2255	1:200, 60 min
		Invitrogen	
VEGF-D	ptLink (pH = 9) Dako	ab63068	1:50, 60 min
		AbCam	
VEGFR-1	ptLink (pH = 9) Dako	ab9540	1:300, 60 min
		AbCam	
VEGFR-2	ptLink (pH = 9) Dako	ab2349	1:100, 60 min
		AbCam	
VEGFR-3	ptLink (pH = 9) Dako	ab72240	1:50, 60 min
		AbCam	

For VEGFs immunohistochemical analyses, the immunohistochemical staining was performed automatically with Ventana Benchmark® XT (Ventana Medical Systems, Tucson, AZ, USA), following manufacturer’s guidelines and then counterstained with hematoxylin and permanently mounted. Negative controls were obtained by omitting the primary antibody incubation step and normal tonsils were used as positive control. Please refer to Table 2 for detailed aspects about each antibody used.

### Immunohistochemical evaluation

Sections were scored semi-quantitatively for expression in cancer cells as follows: 0: 0 % of immunoreactive cells; 1: <5 % of immunoreactive cells; 2: 5-50 % of immunoreactive cells; and 3: >50 % of immunoreactive cells. Also, intensity of staining was scored semi-qualitatively as follows: 0: negative; 1: weak; 2: intermediate; and 3: strong. The final score was defined as the sum of both parameters (extension and intensity), and, for the metabolism-related proteins, grouped as negative (score 0 and 2) and positive (score 3–6), as previously described [27], while, for VEGF family members, the final score was defined as negative (score 0–5) and positive (score 6). Protein expression in the different localizations (cytoplasm, plasma membrane and nucleus) was considered. Two independent observers (ALF and EAG) performed immunohistochemical evaluation blindly and discordant results were discussed in a double-head microscope to determine the final score.

### Statistical analysis

Data were stored and analyzed using the IBM SPSS Statistics software (version 20, IBM Company, Armonk, NY). All comparisons were examined for statistical significance using Pearson’s chi-square (*χ*^2^) test and Fisher’s exact test (when n < 5). The threshold for significant *p* values was established as *p* < 0.05. Overall survival curves were estimated by the method of Kaplan-Meier and data compared using the log-rank test. Cases lacking one or more of the clinicopathological variables were not included in the specific statistical analysis. Also, cases lacking TMA representation (core loss during immunohistochemical procedure or lack of tumor in the TMA core) were excluded from analysis.

## Results

As shown in Table 1, HPV genotyping showed that HPV16 was present in 53.8 % (112/208) of cases, HPV18 was present in 21.2 % (44/208), 11.5 % (24/208) of cases showed infection with other HPV types and 21.6 % (45/208) of cases showed no HPV infection. Also, some cases showed infection by only one HPV type, while others showed infection with 2 or 3 different HPV types.

**Fig. 1:**
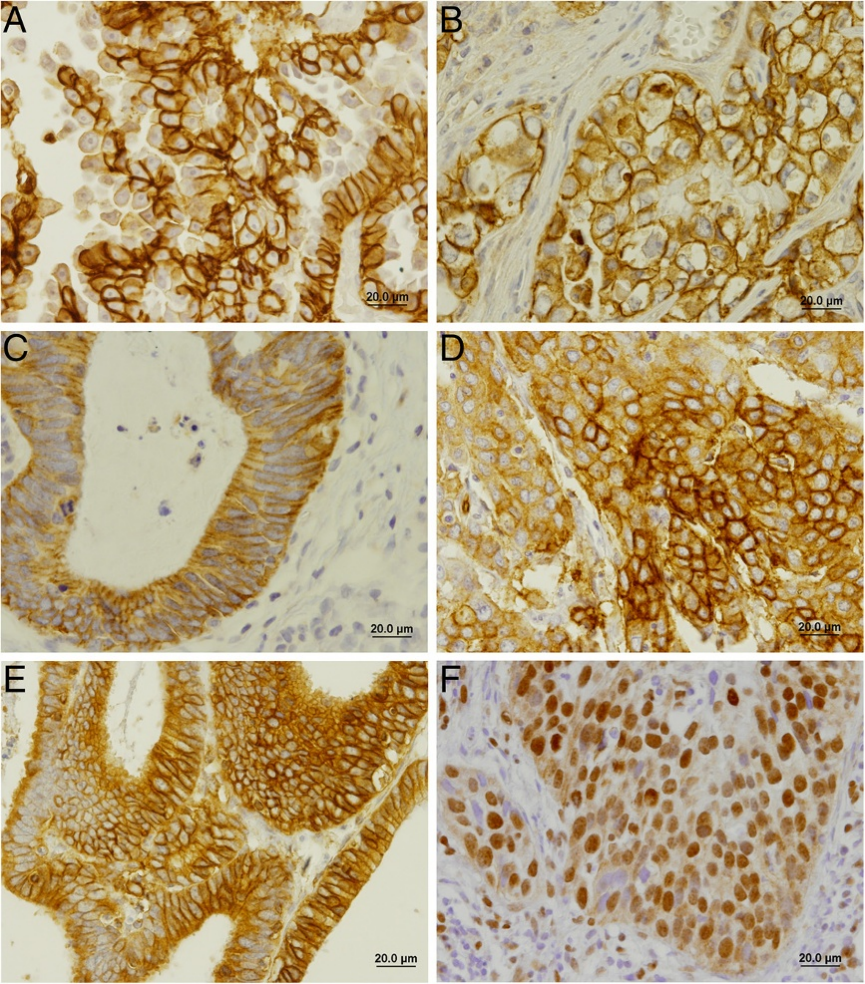
Immunohistochemical expression of MCT1, MCT4, CD147, GLUT1 and CAIX in cervical adenocarcinoma samples. Expression of proteins was more frequently found in the plasma membrane of cells as shown in (a) for MCT1, (b) for MCT4, (c) for CD147, (d) for GLUT1 and (e) for CAIX. Nuclear expression was also observed in some cases for GLUT1 (F)

The immunohistochemical evaluation showed that all the metabolism-related proteins were expressed in the cytoplasm, in the plasma membrane or exhibited both localizations in cancer cells. Interestingly, GLUT1 was also present in the nucleus of 17 out of 197 cases (8.6 %). Fig. 1 shows photomicrographs representative of the expression of each protein. As can be seen in Table 3, MCT4 and CAIX were the proteins more frequently expressed at the plasma membrane of cancer cells (around 85 %), followed by MCT1 (59.2 %), GLUT1 (44.2 %) and, finally, CD147 (6.9 %). When evaluating the co-expression between the proteins (Table 4), an association between MCT4 and CAIX was found (*p* = 0.010).

**Table 3:** Expression frequencies of the studied proteins

	n	Expression Positive (%)
MCT1	196	116 (59.2)
MCT4	202	175 (86.6)
CD147	202	14 (6.9)
GLUT1	197	87 (44.2)
CAIX	192	158 (82.3)
VEGF-A	178	112 (62.9)
VEGF-C	179	162 (90.5)
VEGF-D	176	103 (58.5)
VEGFR-1	171	45 (26.3)
VEGFR-2	176	163 (92.6)
VEGFR-3	168	104 (61.9)

**Table 4:** Associations between the metabolism-related proteins

	CD147			GLUT1			CAIX		
	n	Positive (%)	*p*	n	Positive (%)	*p*	n	Positive (%)	*p*
MCT1			0.409			0.725			0.530
Negative	78	4 (5.1)		76	33 (43.4)		75	61 (81.3)	
Positive	115	10 (8.7)		113	52 (46.0)		112	95 (84.8)	
MCT4			0.695			0.793			0.010
Negative	25	2 (8.0)		21	9 (42.9)		21	13 (61.9)	
Positive	171	12 (7.0)		170	78 (45.9)		169	143 (84.6)	

**Fig. 2:**
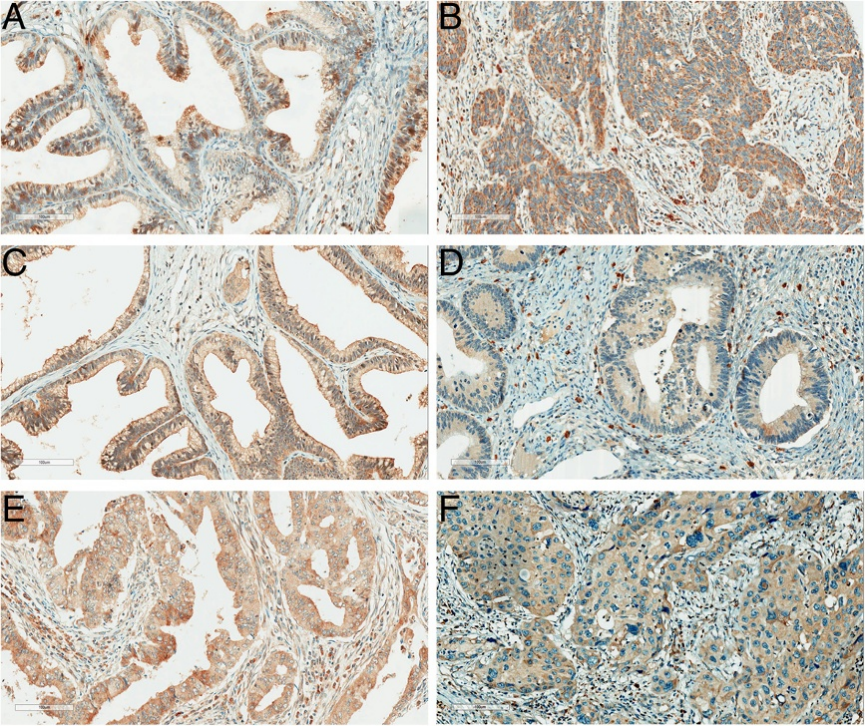
Immunohistochemical expression of VEGF family in cervical adenocarcinoma samples. (**a**) VEGF-A; (**b**) VEGF-C; (**c**) VEGF-D; (**d**) VEGFR-1; (**e**) VEGFR-2; and (**f**) VEGFR-3

Concerning the VEGF family members, overall, these proteins, with the exception of VEGFR-1, presented a high intensity and extension of expression in cancer cells (Fig. 2), which implicated the use of a different cut-off (>5 instead of >2) for the VEGF family in the subsequent analysis. As can be seen in Table 3, VEGF-C and VEGFR-2 were the VEGF family members more frequently expressed in cancer cells (around 90 %), followed by VEGF-A, VEGF-D and VEGFR-3 (around 60 %) and, finally, VEGFR-1 (26.3 %). When evaluating the co-expression between the expression of the ligands and the receptors (Table 5), VEGF-A was more frequently expressed in cancer cells expressing VEGFR-1 or VEGFR-3 (*p* = 0.008 and *p* < 0.001, respectively), VEGF-C was more frequently expressed in cancer cells expressing VEGFR-2 (*p* < 0.001), and VEGF-D was more frequently expressed in cancer cells expressing VEGFR-1 or VEGFR-3 (*p* = 0.002 and *p* < 0.001, respectively).

**Table 5:** Associations between the VEGF family members

	VEGFR-1			VEGFR-2			VEGFR-3		
	n	Positive (%)	*p*	n	Positive (%)	*p*	n	Positive (%)	*p*
VEGF-A			0.008			0.736			<0.001
Negative	63	10 (15.9)		62	58 (93.5)		62	26 (41.9)	
Positive	100	35 (35.0)		98	93 (94.9)		97	72 (74.2)	
VEGF-C			0.183			<0.001			0.281
Negative	12	1 (8.3)		13	8 (61.5)		12	6 (50.0)	
Positive	154	44 (28.6)		148	142 (95.9)		145	95 (65.5)	
VEGF-D			0.002						<0.001
Negative	69	10 (14.5)		70	61 (87.1)		64	28 (43.8)	
Positive	96	35 (36.5)		91	89 (97.8)		91	73 (80.2)	

When evaluating the possible co-expression between the metabolism-related and the VEGF family members (Table 6), significant associations between MCT1 and VEGF-A (*p* = 0.010), and MCT4 and both VEGF-A and VEGFR-3 (*p* = 0.037 and *p* = 0.026, respectively) were found.

**Table 6:** Associations of the metabolism-related proteins with the VEGF family members

	VEGF-A			VEGF-C			VEGF-D			VEGFR-1			VEGFR-2			VEGFR-3		
	n	Positive (%)	*p*	n	Positive (%)	*p*	n	Positive (%)	*p*	n	Positive (%)	*p*	n	Positive (%)	*p*	n	Positive (%)	*p*
MCT1			0.320			0.010			0.958			0.916			0.311			0.686
Negative	61	37 (60.7)		64	53 (82.8)		60	35 (58.3)		57	16 (28.1)		59	54 (91.5)		60	39 (65.0)	
Positive	95	65 (68.4)		96	92 (95.8)		97	57 (58.8)		97	28 (28.9)		93	89 (95.7)		88	60 (68.2)	
MCT4			0.037			0.699			0.878			0.319			0.359			0.026
Negative	21	9 (42.9)		22	21 (95.5)		22	13 (59.1)		22	8 (36.4)		22	22 (100.0)		21	9 (42.9)	
Positive	137	91 (66.4)		140	125 (89.3)		136	78 (57.4)		134	35 (26.1)		134	124 (92.5)		128	87 (68.0)	
CD147			0.138			0.122			0.074			0.187			1.000			0.223
Negative	146	91 (62.3)		149	136 (91.3)		147	82 (55.8)		145	39 (26.9)		143	134 (93.7)		138	89 (64.5)	
Positive	13	11 (84.6)		13	10 (76.9)		12	10 (83.3)		11	5 (45.5)		11	11 (100.0)		12	10 (83.)	
GLUT1			0.718			0.115			0.475			0.387			0.811			0.196
Negative	81	51 (63.0)		83	71 (85.5)		80	43 (53.8)		81	20 (24.7)		81	76 (93.8)		74	46 (62.2)	
Positive	73	48 (65.8)		75	70 (93.3)		74	44 (59.5)		71	22 (31.0)		70	65 (92.9)		72	52 (72.2)	
CAIX			0.876			0.740			0.135			0.982			0.818			0.149
Negative	25	16 (64.0)		26	24 (92.3)		25	11 (44.0)		25	7 (28.0)		26	24 (92.3)		26	14 (53.8)	
Positive	128	84 (65.6)		131	116 (88.5)		128	77 (60.2)		126	35 (27.8)		124	116 (93.5)		118	81 (68.6)	

Finally, the plasma membrane expression of the metabolism-related proteins and the overall expression of the VEGF family members were associated with the available clinicopathological data, with significant results shown in Table 7. For the metabolism-related proteins, the following associations were significant: (i) MCT4 and presence of parametrial invasion (*p* = 0.002) as well as HPV18 infection (*p* = 0.003); (ii) CD147 and presence of distant metastasis (*p* = 0.044); (iii) GLUT1 and presence of distant metastasis (*p* = 0.021); (iv) CAIX and smaller tumor size (*p* = 0.036) as well as HPV18 infection (*p* = 0.004). For the VEGF family members, the following associations were significant: (i) VEGFR-1 and presence of local metastasis (*p* = 0.027); (ii) VEGFR-1 and presence of lymphnode metastasis (*p* = 0.030). Additionally, co-expression analysis showed that the association of GLUT1 expression with presence of distant metastasis was associated with co-expression with MCT1, but not MCT4 (data not shown), while co-expression of GLUT1 with MCT1 was also associated with presence of local metastasis, where 9/21 (42.9 %) cases with local metastasis showed GLUT1/MCT1 co-expression while 14/67 (20.9 %) cases without local metastasis showed GLUT1/MCT1 co-expression (*p* = 0.046). Also, when analyzing the clinicopathological value of the significant co-expressions between metabolism-related proteins and VEGF family members (MCT1/VEGF-C, MCT4/VEGF-A and MCT4 + VEGFR-3), co-expression of MCT4 with VEGFR-3 was significantly associated with parametrial invasion, where 15/20 (75.0 %) cases with invasion showed MCT4/VEGFR-3 co-expression while 5/13 (38.5 %) cases without invasion showed MCT4/VEGFR-3 co-expression (*p* = 0.036). Analysis of survival showed no association with the different proteins studied (data not shown).

**Table 7:** Significant associations between plasma membrane immunoexpression of the proteins analyzed and clinicopathological data

	n	Positive (%)	*p*
*MCT4*			
Parametrial invasion			0.002
Absent	19	12 (66.7)	
Present	26	28 (100.0)	
HPV18 infection			0.003
Negative	143	119 (83.2)	
Positive	39	39 (100.0)	
*CD147*			
Distant metastasis			0.044
Absent	95	3 (3.2)	
Present	17	3 (17.6)	
*GLUT1*			
Distant metastasis			0.021
Absent	96	41 (42.7)	
Present	18	13 (72.2)	
*CAIX*			
Tumor size			0.036
< 4 cm	25	24 (96.0)	
> 4 cm	8	5 (62.5)	
HPV18 infection			0.004
Negative	136	106 (77.9)	
Positive	39	38 (97.4)	
*VEGFR-1*			
Local metastasis			0.027
Absent	51	8 (15.7)	
Present	20	8 (40.0)	
Lymphnode metastasis			0.030
Absent	49	9 (18.4)	
Present	9	5 (55.6)	

## Discussion

Tumor cell metabolic reprogramming and angiogenesis stimulation are included in the well-known hallmarks of cancer [7]. While angiogenesis has been studied for a longer time and is included in the first group of hallmarks of cancer [30], metabolic reprogramming of cancer cells is a much more recent research field. Although some evidence points to a relation between these two hallmarks [13], the crosstalk between metabolism and angiogenesis is still poorly studied. Therefore, in the present study, we characterized the metabolic and vascular profiles of a series of cervical adenocarcinomas, in an attempt to provide additional evidence for the possible crosstalk between these two important characteristics of cancer cells.

The immunohistochemical evaluation revealed a high frequency of expression of MCT4 and CAIX, an intermediate frequency of MCT1 and GLUT1, and a low frequency of CD147. Importantly, the high frequency of MCT4, when compared with MCT1, indicates that this MCT isoform should play an important role in the metabolic reprogramming of cancer cells towards a hyperglycolytic and acid-resistant phenotype. This contribution of MCT4 probably goes in parallel with CAIX activity, as they are significantly co-expressed. In opposition, GLUT1 expression does not account for the hyperglycolytic phenotype of all MCT4/CAIX positive tumors while CD147 does not account for all MCT1 or MCT4 positive tumors. As a result, another glucose transporter as well as another MCT chaperone should also have a role in the metabolic adaptations of cervical adenocarcinoma. The hypothesis of an alternative MCT chaperone has already been raised in other studies [31–34].

Interestingly, we found GLUT1 expression in the nucleus. This is not the first report of GLUT1 in the nucleus [35]; however, to the best of our knowledge, it is the first report in the context of cancer. GLUT1 nuclear expression was found at a low frequency and with no implications in prognosis; however, further studies are required to understand the functional role of GLUT1 in this cellular compartment. GLUT1 nuclear expression presented no association with clinicopathological parameters, but the same was not true for plasma membrane expression of other markers.

MCT4 plasma membrane expression suggests an increase in lactate efflux from cancer cells. As a result, lactate will accumulate in the tumor microenvironment and perform additional roles, including increase in tumor cell motility as well as extracellular matrix remodeling by stimulation of hyaluronan and its receptor CD44, which are molecules involved in the process of cancer invasion and metastasis [13, 36, 37]. These roles of lactate in the extracellular milieu are in accordance with the association of MCT4 expression with presence of parametrial invasion. Besides the important role of extracellular lactate, we should not forget that MCTs interact with CD147 at the plasma membrane. This protein has been firstly described thanks to its capability of increasing the production of matrix metalloproteinases (MMP), having a role in tumor invasion and metastasis [38]. As a result, by interacting with CD147 at the plasma membrane, as well as contributing for CD147 maturation and trafficking to the plasma membrane [39, 40], MCTs are additionally involved in cancer invasion and metastasis. In accordance with this role of CD147, in the present study, we found a significant association of this protein with presence of distant metastasis. Previous studies have shown association of CD147 with lymphnode metastasis in cervical cancer [41, 42] but, to the best of our knowledge, this is the first study showing association with distant metastasis in cervical cancer. Following the rationale that hyperglycolytic and acid-resistant tumors are associated with a more aggressive phenotype [43], associations of GLUT1 and CAIX with poor prognostic variables are expected. In fact, we found GLUT1, alone or in combination with MCT1, but not MCT4, to be associated with presence of distant metastasis. Although GLUT1 has been previously associated with lymphnode metastasis in cervical cancer [44], to the best of our knowledge, this is the first report associating GLUT1 with distant metastasis in cervical cancer. This finding contradicts another study in locally advanced cervical carcinoma, where absence of GLUT1 was shown to significantly increase the likelihood of metastasis-free survival [45]. It is important to highlight, however, that the latter study was performed in a tumor series containing only squamous cell carcinomas. Although MCT4 is more frequently expressed in cervical adenocarcinomas than MCT1, MCT1 also seems to have an important role in the metabolic remodeling and aggressive behavior of these tumors as, besides being associated with presence of distant metastasis when co-expressed with GLUT1, MCT1 co-expression with GLUT1 is also associated with presence of local metastasis. In fact, in a previous study, we show that, in cervical adenocarcinomas, MCT1 co-expression with CD147 is associated with presence of metastasis (lymphnode and distant metastasis) [46]; however, in the present study, only co-expression with GLUT1 showed significant associations. Concerning CAIX, previous studies on the clinicopathological significance of this pH regulator in cervical cancer showed association with presence of distant metastasis [47], lymphnode metastasis, advanced tumor stage, greater invasion depth and higher tumor grade [48]. In the present study, these associations were not found, probably because we focused only in adenocarcinomas, while the former studies considered tumors series containing the different histological subtypes, with a majority of squamous cell carcinomas. However, other associations were found for CAIX in the present study. CAIX was more frequently expressed in smaller tumors, probably as a response to initial tumor hypoxia (as CAIX is a target of hypoxia-inducible factor (HIF) 1α [49]), before establishment of the new blood vessel network.

Importantly, we found significant associations between HPV18 infection and both MCT4 and CAIX. Although a previous work found no association between CAIX expression and HPV *status* in head and neck squamous cell carcinomas [50], MCT4 expression has been previously associated with high-risk HPV in cervical cancer [31]. CAIX and MCT4 have the common feature of being HIF-1α-induced key metabolic proteins [49, 51], and HPV infection has been demonstrated to stabilize HIF-1α expression [52, 53]. Therefore, HPV may, indirectly through HIF-1α, have a role in the metabolic reprogramming of cancer cells, by stimulating, among other HIF-1α targets, MCT4 and CAIX, key proteins in the hyperglycolytic and acid-resistant phenotype of cancer cells. Importantly, HIF-1α was recently described as a predictor of poor prognosis in cervical cancer [54]. Further studies are required to elucidate the specific mechanisms involved in this possible stimulation since GLUT1, another HIF-1α target [55], was not associated with HPV18 in this tumor series.

As mentioned above, lactate, besides being the product of the predominant metabolic pathway shown by the majority of cancer cells, has important roles in the tumor microenvironment, contributing to the malignant behavior of cancer cells [13]. One additional role of exogenous lactate is the induction of VEGF [56], contributing to the angiogenic switch characteristic of tumor progression. Following this line of evidence, we analyzed the expression of VEGF family members in the cervical adenocarcinomas samples and verified its co-expression with the metabolism-related proteins. Firstly, we observed an overall high expression of these proteins, especially VEGF-C and VEGFR-2. This ligand/receptor pair may function together to stimulate either angiogenesis or lymphangiogenesis [20], so this result is in accordance with previous publications, where intense angiogenesis in cervical adenocarcinomas [57] and high expression of VEGF family members in cervical cancer is described [58]. When analyzing the co-expression of the metabolism-related proteins with the VEGF family members, we found associations between MCT1 and VEGF-C as well as MCT4 and both VEGF-A and VEGFR-3. These findings support the previous study showing VEGF stimulation by lactate [56], supporting the hypothesis of a crosstalk between the metabolic remodeling of cancer cells and the angiogenic switch. In respect to VEGF family members’ clinicopathological significance, some previous studies show the clinicopathological value of VEGF family members in cervical adenocarcinoma, as VEGF-A, VEGF-C and VEGFR-3 were associated with lymphnode metastasis [58, 59]. Similarly, in the present study, we found VEGFR-1 to be more frequently expressed in cases with local metastasis or lymphnode metastasis, supporting the value of VEGF family members in prognosis. Finally, the association of MCT4 with parametrial invasion was maintained when co-expressed with VEGFR-3, indicating a probable cooperation between metabolism and angiogenesis to enhance cancer cell aggressiveness.

## Conclusions

In the present study, we found evidence pointing at a crosstalk between two important hallmarks of cancer: deregulating cellular energetics and inducing angiogenesis. Additionally, we reinforced the contribution of these hallmarks to cancer progression and aggressiveness, and point at promising therapeutic targets for cancer therapy, such as MCTs. Further studies are warranted to confirm these findings as well as characterize the metabolic and vascular profile of other tumor types.

## Abbreviations

CAIX: Carbonic anhydrase IX
GLUT1: Glucose transporter 1
HPV: Human papillomavirus
HIF-1α: Hypoxia inducible factor 1 alpha
MCTs: Monocarboxylate transporter
TMA: Tissue microarray
VEGF: Vascular endothelial growth factor
VEGFR: Vascular endothelial growth factor receptor
